# Blood donation practice and its associated factors among health professionals of University of Gondar Hospital, Northwest Ethiopia: a cross sectional study

**DOI:** 10.1186/s13104-017-2618-5

**Published:** 2017-07-19

**Authors:** Getachew Arage, Seada Ibrahim, Endeshaw Adimasu

**Affiliations:** 1 Department of Nursing, College of Health Sciences, Debre Tabor University, Debre Tabor, Ethiopia; 2 Department of Midwifery, College of Medicine and Health Sciences, University of Gondar, Gondar, Ethiopia; 3 Department of Nursing, College of Medicine and Health Sciences, University of Gondar, Gondar, Ethiopia

**Keywords:** Blood donation, Practice, Health professionals, Ethiopia

## Abstract

**Background:**

Blood donation has remained a challenge in developing countries, like Ethiopia. In Ethiopia there is a high reliance on family surrogate and waged blood donors which carries an attendant increased risk of transfusion transmissible infection. Health workers are expected to practice blood donation so as to create a good image to the public. A study on blood donation behavior may improve successful implementation of the blood donation programs.

**Methods:**

An institution based cross-sectional study was deployed from January to June 2015. An aggregate of 427 health workers were included in the study by using simple random sampling technique. Data were collected by using pre tested and structured questionnaire via self-administrated method. Descriptive and summary statistics were employed. Bivariate and multiple logistic regressions were computed. Odds ratios and their 95% confidence intervals were calculated to determine the level of significance.

**Results:**

A total of 427 participants were included in the final analysis (response rate = 100%). Among these participants, 33.2% of them practice blood donation. Age above 25 years [AOR = 1.8 (95% CI 1.1, 3.0)], health professionals’ knowledge of blood donation [AOR = 1.9 (95% CI 1.1, 3.1)], health professionals’ attitude towards blood donation [AOR = 3.0, 95% CI 1. 8, 4.9)], and the presence of family members or relatives who received blood [AOR = 5.4, 95% CI 3.7, 8.7)] were significantly and independently associated with blood donation behavior of health professionals.

**Conclusions:**

Blood donation practice of health professionals in this study was found to be low as compared to other studies conducted in developing countries. Health professionals’ knowledge, attitude, age and the presence of family members or relatives who received blood before were independently associated with blood donation practice. Thus, awareness has to be created for health professionals to improve blood donation practices.

## Background

Blood is an essential element of human life and there are no substitutes for it [1]. World Health Organization (WHO) proposes countries to focus on young people to achieve 100% non-remunerated voluntary blood donation by 2020 Dhingra [2]. Donated blood can be life saving for individuals who have lost large volumes of blood from serious accidents, obstetric and gynecological hemorrhages, or surgery and stem cell transplant patients as well as for individuals who have symptomatic anemia from medical or hematologic conditions or cancers. Therefore, blood is an important concern to the society. The use of these lifesaving products may be complicated by infectious and immunological diseases some of which could be life threatening [3].

The blood donation is the only source of blood but the recruitment of voluntary, non-remunerated donors is the most important challenge throughout the world [4]. Blood is the essence of life, and is one of the most precious donations. Blood services are facing shortage of blood all over the world. Demand for blood is rising alarmingly and current blood donation is insufficient to meet the demand [5]. The only source of blood is by blood donation [6]. Globally, 80 million units of blood are donated each year, but only two million units are donated in sub-Saharan Africa where the need is very high [7]. In sub-Saharan Africa (SSA), out of the estimated need of 18 million units of safe blood per year, merely about 15% were collected [8].

Adequate and safe blood supply has remained a challenge in developing countries like Ethiopia [9]. In Ethiopia, there has been gross inadequacy and in equitability in access to blood. The national requirement for blood in Ethiopia is between 80,000 and 120,000 units per year but only 43% is collected [10].

Blood banks have a duty to provide adequate and safe blood to the community. Generally, donors are classified as: voluntary, family replacement remunerated or paid donors, and autonomous donors. The risk of transfusion transmissible diseases is highest with the use of blood procured from remunerated donors. A person who is in need of money is more likely to hide his/her true state of health [3].

Many studies have been conducted to determine people’s knowledge, attitude and practice of blood donation. However, to be motivated or discouraged about blood donation is still challenging.

Moreover, it should be understood that blood supplies are sufficient, in balance with demand and are gathered from low-risk population. For this reason, health workers are expected to practice blood donation so as to create a good image to the public [3, 11]. Hence, the study was aimed at assessing factors and blood donation practices among health professionals.

## Methods

An institution based cross-sectional study was conducted from January to June 2015 among health professionals at University of Gondar Hospital in Northwest Ethiopia. University of Gondar Hospital is found in Gondar town which is 748 km apart from Addis Ababa, the capital city of Ethiopia. The hospital is the oldest medical school in Ethiopia. It was established as the Public Health College in 1954. It is a referral hospital for four district hospitals in the area and is serving more than 5 million people. It has also a blood bank which is established before 10 years. The hospital has 550 beds and 822 health professionals. Out of 822 health professionals, 186, 493, 71, 51, 9, 12 are physicians, nurses, laboratory science technologists, pharmacists, anesthetists and radiographers respectively. The hospital renders services for about averagely 500 clients per day. Annually, about 219,968 clients get services in the hospital.

All health professionals working in University of Gondar Hospital and those available during the study period were the source population and study population respectively.

### Sample size determination

The sample size was calculated by considering the assumptions for single population proportion formula: the proportion (P) = 50%, Z = standard normal distribution value at 95% confidence level of Za/2 = 1.96, 5% of absolute precision, and 10% non-response rate. Hence, the total sample size was 427. Simple random sampling technique was used to select the study participants. Proportional allocation was employed to select the study participants. There are a total of 822 health professionals. When they are divided proportionally; 97 physicians, 256 nurses, 37 laboratory science technologists, 26 pharmacists, 5 anesthetists and 6 radiographers were taken.

### Data collection procedures

Pre-tested and structured questionnaires using self-administered were used for data collection. The questionnaire was adapted after reviewing similar literatures [5, 7, 8]. A total of 42 items were used to answer the objectives of the study. The questionnaire was divided into four sections. These sections were Socio-demographic characteristics, Knowledge on blood donation, attitude towards blood donation and blood donation practice. Pre-testing of the questionnaire was undertaken on 22 health professional (5% of the sample size) in Debre Tabor General Hospital which is a nearby hospital. Some items in the questionnaire were revised for clarity, wordiness, logical sequence, and skip pattern after the pre-test was done. Data collection was facilitated by two facilitators who hold bachelor degree in nursing in University of Gondar Hospital. The facilitators were selected based on their previous experiences. A 2-day comprehensive training was given for the facilitators.

The questionnaires were coded and entered into EPI Info version 3.5.3 statistical software and then exported to SPSS windows version 16 for further analysis. Data were summarized and presented using descriptive statistics. Bivariate and multiple logistic regressions were computed to identify the presence and strength of associations. Odds ratios with 95% CI were computed and variables having P values less than 0.05 in the multiple logistic regression models were considered significantly associated with the dependent variable.

### Operational definition

*Knowledge about blood donation* was categorized after asking 9 questions. Those respondents who scored greater than or equal to the mean for knowledge questions were considered as knowledgeable, otherwise not.

*Attitude about blood donation* was categorized after asking 8 questions using a likert scale method and the mean scores were computed and dichotomized into favorable (score ≥ mean value) or unfavorable (<mean value).

*Blood donation practice* in this study was considered if the respondents had ever donated blood at least one time in life.

*Current donors* If the time period of donating blood is less than 1 year.

*Elapsed donors* If the time period of donating blood is 1–2 years.

*Never donors* If the participants did not donate blood in his/her life.

## Results

### Socio-demographic characteristics of the study participants

A total of 427 health professionals of University of Gondar Hospital have participated in the study making a response rate of 100%. This 100% response rate was achieved since the data collection facilitators were experienced and the issues of blood donation are not sensitive. As a result, the participants were not failing to comply with the intended study. The mean age of the study participants was 26 years (±SD = 5.13). The majority 293 (68.6%) of the participants were males. Majority of the participants 369 (86.4%) were Orthodox Christians, and were from Amhara ethnic group 366 (85.7%). More than half of the participants 249 (58.3%) were from nursing department. 284 (66.5%) were bachelor degree (BSc) holders (Table 1).

**Table 1:** Socio-demographic characteristics of the study participants, University of Gondar Hospital, Northwest Ethiopia, April, 2014 (n = 427)

Variable	Frequency	%
Age (n = 427) (years)		
<25	170	39.7
≥25	257	60.3
Sex (n = 427)		
Female	134	30.4
Male	293	69.6
Religion (n = 427)		
Orthodox	369	86.4
Muslim	41	9.4
Others^a^	17	4.2
Educational status (n = 427)		
Diploma	19	4.5
Bachelor (BSc)	284	67.5
Masters (MSc)	22	5.3
General practitioners (GP)	92	21.5
Specialists	10	1.2
Department (n = 427)		
Nursing	249	58.5
Pharmacy	27	6.4
Medicine	95	22.3
Laboratory	38	9.2
Radiology	6	1.6
Anesthesia	12	2.0

^a^Protestant and catholic

### Knowledge and attitude about blood donation

Two hundred and thirteen (49.8%) participants were knowledgeable about blood donation, but about half 218 (51.6%) the respondents had unfavorable attitude.

### Blood donation practice

One hundred forty two (33.2%) of the respondents reported that they have ever donated their blood at least one time. To the contrary, 63 (44.3%) of the participants replied as they have never donated at all. Eighty of the respondents (56.3%) who had ever donated blood replied that they feel comfortable after donating blood. Seventy-three (17.9%) of the total respondents said that one or more members of their family had received blood at least once. The main source of blood being from other family member in 36 (49.3%) followed by Ethiopian Red Cross Society, 30 (41.1%) (Table 2).

**Table 2:** Distribution of the study participants in relation to practice of blood donation, University of Gondar Hospital, Northwest Ethiopia, April, 2014 (n = 142)

Variable	Frequency	%
Number of times donated (n = 142)		
Once	70	49.3
Twice	43	30.3
Three times or more	29	20.4
Time after the last donation (n = 142)		
Current donors	47	33.1
Elapsed donors	31	23.2
Never donors	63	43.7
Feeling after donating blood		
Comfortable/positive	80	56.3
Some discomfort/indifferent	62	43.6
Family member ever received blood (n = 427)		
No	349	82.4
Yes	78	17.6
Source of the blood received by the family member (n = 73)		
Other family member/relative	36	49.3
Ethiopian Red Cross Society	30	41.1
Other sources^a^	7	9.6

^a^From other volunteer donors, some receives money as compensation

### Reasons for not donating blood

**Fig. 1:**
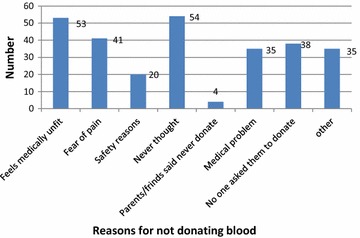
Reasons for not donating blood among Health Professionals at University of Gondar referral Hospital, Northwest Ethiopia, April, 2014 (n = 280). The commonly mentioned reasons for not donating blood were: fear of pain (21.42%), never thought about blood donation (19.21%), feels physically unfit (18.92%), no one asked about blood donation (17.4%) and medical problems (16.1%)

The main reasons given by the respondents for not donating blood was found to be their inability to think about blood donation 54 (19.2%) and their feeling that they are medically unfit 53 (18.9%) (Fig. 1).

### Factors associated with practice of blood donation

This study revealed that, age of the respondents, knowledge about blood donation, attitude towards donating their blood and having any family member ever received blood were significantly associated with practice of blood donation in bi-variable and multivariable logistic regression analysis.

Participants whose family members had received blood were five times more likely to donate blood as compared to those whose family members had not received blood (AOR = 5.4, 95% CI 3.7, 8.7). Those who were 25 years old were about two times more likely to donate blood than those who are 25 years old or less (AOR = 1.8, 95% CI 1.1, 3.0). Respondents who scored more than the mean of the knowledge questions about blood donation were about two times more likely to donate blood when compared to those who scored the mean result or less (AOR = 1.9, 95% CI 1.1, 3.1). Participants who had favorable attitude to donate their blood were three times more likely to donate blood when compared to these who had unfavorable attitude to donate their blood (AOR = 3.0, 95% CI 1. 8, 4.9) (Table 3).

**Table 3:** Bi variable and multivariable analysis of factors associated with practice of blood donation among health workers of University of Gondar Hospital, Northwest Ethiopia, April, 2014 (n = 427)

Variables	Ever donated blood		OR (95% CI)	
	Yes	No	COR (95% CI)	AOR (95% CI)
Age (years)				
<25	126 (74.3%)	44 (26.7%)	1	1
>25	159 (61.8%)	98 (38.1%)	1.7 (1.1, 2.7)	1.8 (1.10, 3.00)
Knowledgeable				
Yes	90 (40.5%)	132 (59.5%)	1.94 (1.28, 2.93)	1.9 (1.1, 3.10)
No	57 (27.8%)	148 (74.0%)	1	1
Attitude towards donating their blood				
Favorable	94 (46.1%)	110 (53.9%)	3.02 (1.98, 4.62)	3.0 (1.1, 4.9)
Unfavorable	53 (23.7%)	170 (78.0%)	1	1
Family member ever received blood				
Yes	61 (83.6%)	17 (21.7%)	6.82 (5. 3, 9.7)	5.4 (3.7, 8.7)
No	81 (23.2%)	268 (76.8%)	1	1

## Discussion

The finding from this study has showed that 33.2% (95% CI 28.5, 37.5) of the respondents have ever donated blood prior to the study period. This finding is somewhat similar with the findings of a cross-sectional study conducted among Yazd community in Iran where 38% of the respondents had ever donated blood [12]. However, the result of this study is relatively high as compared to the findings of the studies done in Trinidad and Tobago [13], Dhaka in Bangladesh [14], India [15], and Nigeria [7], where the practice of blood donation among the study participants was 18.8, 16, 10.8, and 15.3% respectively.

Nevertheless, the finding of this study was relatively low as compared to the findings of a study in Saudi Army force hospital where 58.2% of the study participants have ever donated blood [16]. This could be due to the defiance in socio-cultural background, difference in study areas, for instance, in the Saudi Army Hospital; the possibility of donating blood is expected to be high as blood could frequently be needed by the army casualties. Activities of organization working in blood bank could be one reason for the difference in the proportion of the study participants who had ever donated blood. In Ethiopia, Ethiopian Red Cross Society and other health associations like Ethiopian Public Health Association, Ethiopian Society of Obstetricians and Gynecologists and Ethiopian Midwives Association are currently promoting blood donation in collaboration with Ethiopian Federal Ministry of Health and other international organizations which might have increased the awareness and might have changed the attitude of the respondents towards donating blood.

A number of factors were reported as predictors of blood donation behaviors of health professionals. In the present study, the age of the study participants was significantly associated with practice of blood donation. Participants who were more than 25 years old were two times more likely to donate blood when compared to those who are 25 years old or less. This is in agreement with a study done in Maryland in USA [17] where older age group were about two times more likely to donate blood when compared to the younger ones. This might be due to the fact that older people have faced different problems in their relatives or families. This could have changed the attitude of their friends (usually in the same age group) to donate blood.

Knowledge of the study participants about blood donation is another factor which was significantly associated with the practice of blood donation. Those who were knowledgeable (scored above mean score of the knowledge questions) were about two times more likely to donate blood when compared with those scored mean score or less. This can be explained by the fact that those who are knowledgeable about blood donation know that donating blood does not harm the donor and hence could have donated the blood to save lives.

Having favorable/positive attitude towards donating their own blood was also one of the factors which were significantly associated with practice of blood donation. Those study participants who have maintained favorable attitude towards donating their blood (scored above mean score of the attitude questions) were three times more likely to donate blood when compared with those who have negative/unfavorable attitude towards donating their blood. This is in line with a study conducted in Maryland in USA [17] where attitudinal problems were mentioned as a hindering factor for blood donation.

This can be explained by the fact that if someone has negative attitude towards something, he/she will not practice it. Feeling that they are medically unfit, fear of pain during drawing blood sample, pressure from their relatives/families or friends not to donate blood, and fear of safety during the donation time were among the main reasons the respondents mentioned for having unfavorable attitude towards blood donation. This could also be attributed to inadequacy of knowledge about blood in general.

Having a family member or relative who had ever received blood was another factor which was significantly associated with the practice of blood donation among the study participants. Those study participants whose family members or relatives had ever received were about five times more likely to practice blood donation.

This could be because those study participants whose family member(s) or relative(s) were in need of blood to continue their life have donated blood might have practiced it for the first time even while their attitude towards donating their blood is negative for the sake of saving the life of their beloved ones. Having once practiced blood donation and feeling that it is not such much posing discomfort without the pain they feel during needle piercing their vein during the drawing but saving the lives of their relatives could have changed the attitude of those participants so that they would have repeated the donation after that even for non-relative ones.

However, the present study has some inherent limitations. The cross-sectional nature of the study which used a snapshot of prevalence of blood donation practice may hinder the cause and effect relationship. This kind of study is better supported by qualitative studies especially to assess attitude of the respondents towards blood donation and this study lacks the qualitative part.

## Conclusions

Blood donation practice of health professionals in this study was found to be low as compared to other studies conducted in developing countries. Health professionals knowledge, attitude, age and having family members or relatives received blood before were independently associated with blood donation practice. Thus, creation of awareness about blood donation should be encouraged among health professionals.

## Abbreviations

AOR: adjusted odd ratio
EPI info: a free public domain software package developed by the Centers for Disease Control
ERC: Ethical Review Committee
SD: standard deviation
SPSS: statistical package for social sciences
SSA: sub-Saharan Africa
WHO: World Health Organization
